# ﻿A new systematic arrangement for the blister beetle genus *Eurymeloe* (Meloini, Meloidae, Coleoptera) with the description of a new species from Spain

**DOI:** 10.3897/zookeys.1109.83863

**Published:** 2022-07-01

**Authors:** Alberto Sánchez-Vialas, José L. Ruiz, Ernesto Recuero, Felipe Gutiérrez-Pérez, Mario García-París

**Affiliations:** 1 Museo Nacional de Ciencias Naturales (MNCN-CSIC), calle José Gutiérrez Abascal 2, 28006, Madrid, Spain Museo Nacional de Ciencias Naturales Madrid Spain; 2 Instituto de Estudios Ceutíes, Paseo del Revellín 30, 51001 Ceuta, Spain Instituto de Estudios Ceutíes Ceuta Spain; 3 Department of Plant & Environmental Sciences, Clemson University, Clemson, SC 29634, USA Clemson University Clemson United States of America; 4 Avenida Francisco Javier Sauquillo 22, 28944, Madrid, Spain Unaffiliated Madrid Spain

**Keywords:** *Bolognaia* subgen. nov., *Coelomeloe*, Iberian Peninsula, new species, phylogeny, systematics

## Abstract

The taxonomic status and subgeneric arrangement of the genus *Eurymeloe* have been debated for decades. In this work, the internal taxonomy of *Eurymeloe* is redefined by recognising three subgenera: *Eurymeloe* for the former *Eurymeloebrevicollis* species group, *Coelomeloe* for *Eurymeloetuccia*, and *Bolognaia* Ruiz, García-París, Sánchez-Vialas & Recuero, **subgen. nov**., to accommodate the species of the formerly recognised *Eurymeloerugosus* species group. Additionally, a new species of the newly described subgenus Bolognaia is described from the Iberian Peninsula based on molecular and morphological traits. The new species, Eurymeloe (Bolognaia) orobates**sp. nov.**, can be distinguished from all other species of *Eurymeloe* by the following combination of morphological traits: dispersed brownish setae over the body that are arranged in small tufts on the abdominal terga; a small, very transverse pronotum that presents a unique macrosculpture; a deeply and densely punctured integument of the head and pronotum; and the very rugose elytra. The characters displayed by *E.orobates* suggest that the species groups that were previously defined and recognised for *Eurymeloe*, and that are now integrated within the newly erected subgenus Bolognaia, are non-monophyletic.

## ﻿Introduction

The taxonomy of Eurymeloe Reitter, 1911, originally described as a subgenus of Meloe Linnaeus, 1758, has been controversial due to both its differential use at the genus (Selander 1985; Sánchez-Vialas et al. 2021) or the sub-genus level (see Bologna 1988, 1991, 2008, 2020a; Bologna et al. 1989; Bologna and Pinto 2001; García-París et al. 2010) and its relationship with the monospecific *Coelomeloe* Reitter, 1911, which has been considered a synonym of *Eurymeloe* (Selander 1985; Bologna 2020b; Sánchez-Vialas et al. 2021) or as a separate but closely related subgenus (Bologna 1988, 1991; Bologna et al. 1989; Bologna and Pinto 2001; Di Giulio et al. 2013). Based on morphology, *Eurymeloe* was defined and recognised as two clearly diagnosable species groups, “*E.brevicollis*” and “*E.rugosus*” [Bologna, 1988; as Meloe (Eurymeloe)]. However, according to recent molecular phylogenetic analyses (Sánchez-Vialas et al. 2021), *Coelomeloe* (a former subgenus of *Meloe*) and the species of the “*E.brevicollis* species group” sensuBologna (1988) constitute a monophyletic assemblage that is the sister clade of the “*E.rugosus* species group” (sensuBologna 1988). These analyses have highlighted that, although the generic taxonomic status of *Eurymeloe* is well supported by molecular and morphological data (Selander 1985; Sánchez-Vialas et al. 2021), its internal taxonomic structure remains in need of reassessment.

The *Eurymeloerugosus* species group was comprehensively revised by Bologna (1988), who established two main subgroups based on several morphological traits. The first one, subgroup A or the “*E.rugosus* subgroup”, was formed by species presenting black (or partially brownish) pilosity over the body, an opaque or shiny black integument, deep punctures on the head and pronotum, and rugose elytra. The species forming the second one, subgroup B or the “*E.murinus* subgroup”, were characterised by having a general brownish yellow pilosity, greyish black or brownish black (exceptionally pale brown) integument with a matte or satin appearance, shallow punctures on the surface of the head and pronotum, and elytra that are less rugose than those of the first subgroup. In addition to these two subgroups, Ruiz et al. [2010; as Meloe (Eurymeloe)] proposed a third one, the “*E.saharensis* subgroup”, which includes *Eurymeloesaharensis* (Chobaut, 1898) (widely distributed from the Canary Islands to the Arabian Peninsula) and a closely related species, *E.vignai* (Bologna, 1990). Compared to the first two subgroups, this last one presents distinctive morphological characters such as a slender appearance, long antennae and legs (especially tarsi), silky black integument, pilosity consisting of short reddish setae, thick but sparse punctures on the head and pronotum, and a very shallow elytral rugosity. To date, ca. 20 Palaearctic species have been included in the *E.rugosus* species group (Bologna 1988; Ruiz and García-París 2009, 2015; Ruiz et al. 2010). However, very few studies have examined the phylogenetic relationships among them.

Recently, Sánchez-Vialas et al. (2021) showed that the clade corresponding to the *E.rugosus* species group is comprised of at least three main lineages: one represented by *Eurymeloefernandezi* (Pardo Alcaide, 1951) with the other two corresponding generally to Bologna’s (1988) subgroups A (*E.rugosus* subgroup) and B (*E.murinus* subgroup). However, as pointed by Ruiz and García-París (2009), the morphological traits defining these groups require further study as the specific composition of each is unclear. Moreover, although Sánchez-Vialas et al. (2021) included several representatives of Bologna’s (1988) two subgroups, their study lacked specimens of *E.rugosus* (Marsham, 1802), i.e., the primary species characterising subgroup A, as well as those of the *M.saharensis* subgroup.

We recently collected specimens representing a new distinctive species of the *E.rugosus* species group from central Spain. The new species, which can be easily diagnosed by both conspicuous morphological characters and molecular data, is characterised by a combination of morphological traits present in either one or the other of Bologna’s (1988) subgroups, suggesting that these subgroups are not monophyletic and that some of the characters used to distinguish them (e.g., body setae colouration) are not diagnostic.

In this study, we (1) describe the new species of *Eurymeloe* and analyse its phylogenetic relationships by using the Meloini molecular framework of Sánchez-Vialas et al. (2021), together with other published (Rulik et al. 2017; Ohnishi et al. 2021) and unpublished data, including sequences of *E.rugosus*; (2) describe a new subgenus within *Eurymeloe*, Bolognaia subgen. nov., to accommodate the species of the *E.rugosus* species group (sensuBologna 1988, 1991); (3) redefine, in a more restricted sense, the subgenus Eurymeloe; and (4) discuss the internal taxonomic structures within the new subgenus.

## ﻿Materials and methods

We studied the external morphology of a total of 326 specimens belonging to 17 species of *Eurymeloe*. All specimens are listed in either Ruiz and García-París (2009, 2015) or Sánchez-Vialas et al. (2021), and housed in collections at the Museo Nacional de Ciencias Naturales (**MNCN-CSIC**), Madrid, Spain; the Museu de Zoologia, Barcelona, Spain (**MZB**); and the Natural History Museum, London, England (**NHMUK**); or in the personal collections of M. A. Bologna, University “Roma Tre”, Rome, Italy (**MAB**); and J. L. Ruiz, Ceuta, Spain (**JLR**). A specimen from Biel (Zaragoza) recorded as *Meloerugosus* (currently *Eurymeloerugosus*, see Sánchez-Vialas et al. 2021) by Recalde et al. (2002) and Pérez-Moreno et al. (2003) and housed in the entomological collection of the Sociedad Entomológica Aragonesa (**SEA**) [Maynar-Duplá Collection], Zaragoza, Spain, was also studied. In addition, we examined specimens of *Eurymeloegomari* (Ruiz and García-París 2009) from Morocco, *E.ganglbaueri* (Apfelbeck, 1907) from Sardinia (Italy), and a new species of *Eurymeloe* from central Spain. Comparisons with the remaining species included in Bologna’s (1988)*E.rugosus* species group (i.e., those distributed in the Middle East and the eastern Mediterranean) were made using diagnostic morphological traits extracted from the literature (mainly Kaszab 1958, 1983; Bologna 1988, 1991; Ruiz and García-París 2009, 2015; Ruiz et al. 2010). The geographic distributions of the studied species were also extracted from the literature, mostly from studies by Bologna (1988, 1991, 2008, 2020a).

The description of the new species of *Eurymeloe* is based on a total of five specimens (one male, dried preserved, and four females, ethanol-preserved), all belonging to the type series. These specimens were collected from the mountains of central Spain, at Puerto de la Quesera (Province of Guadalajara, Autonomous Community of Castilla-La Mancha). The type series is held at MNCN-CSIC.

For the morphological study, dry-mounted specimens were examined under a stereomicroscope. The male specimen was rehydrated in water before the extraction of the genital structures, which was subsequently mounted on a piece of cardboard using dimethylhydantoin formaldehyde (DMHF) resin and pinned adjacent to the specimen. Measurements were taken using a micrometre that was coupled to one of the microscope eyepieces. Digital images of live, dry-mounted specimens, and of male and female genital structures, were taken with a reflex camera (Canon 77D) fitted with a macro-lens (Sigma 105 mm F2.8) and two external flashes. We used the terminology suggested by Selander (1966) to describe the various parts of the male genitalia.

For the molecular analyses, we used sequences of *Eurymeloe* available from GenBank and those of four new specimens that we had collected and preserved in ethanol (now housed at the MNCN-CSIC), including one from Morocco corresponding to *Eurymeloegomari*, one from Sardinia (Italy) corresponding to *E.ganglbaueri*, and two from central Spain corresponding to the new species, *Eurymeloe* sp. nov. From GenBank, we downloaded the sequences of 31 specimens belonging to 13 species of *Eurymeloe* (Rulik et al. 2017; Ohnishi et al. 2021; Sánchez-Vialas et al. 2021) (Table 1). A total of 31 specimens of 16 species from ten genera of Meloidae was used as the outgroup (Sánchez-Vialas et al. 2021) (Table 1). Tissue sampling, DNA extraction, sequencing, and alignments were performed as described by Sánchez-Vialas et al. (2021).

**Table 1.** Specimen identity, collection locality, voucher number, and GenBank accession numbers of the new and previously published sequences analysed in this work.

Taxon	Locality	Voucher number	GenBank # CoxI	GenBank # 16S	GenBank # Wg	GenBank # 18S
*Eurymeloeapivorus*	Morocco: Fès-Meknès: Ifrane, 2 km South of Cedro Gouran, Middle Atlas	**mel 81009**	MW158218	MW158046	MW157964	MW158119
*Eurymeloeapivorus*	Morocco: Fès-Meknès: Ifrane, 2 km South of Cedro Gouran, Middle Atlas	**mel 81013**	MW158220	MW158048	MW157966	MW158121
*Eurymeloeapivorus*	Morocco: Fès-Meknès: Ifrane, 2 km South of Cedro Gouran, Middle Atlas	**mel 81054**	MW158219	MW158047	MW157965	MW158120
*Eurymeloebrevicollis*	Spain: Cantabria: Brañavieja, Pico Tres Mares	**mel 04107**	MW158305	MW158088	MW157987	MW158142
*Eurymeloebrevicollis*	Andorra: Arinsal	**mel 07092**	MW158306	MW158089	MW157988	MW158143
*Eurymeloecorvinus*	Japan: Niigata, Sado, Kitaushima	**9060**	LC583106.1			
*Eurymeloecorvinus*	Japan: Saga, Karatsu, Hirose	**11881**	LC583105.1			
*Eurymeloecorvinus*	Japan: Nagano, Iriyamabe	**10521**	LC583104.1			
*Eurymeloefernandezi*	Spain: Islas Canarias: La Palma, Los Sauces	**mel 07045**	MW158266	MW158068	MW157972	MW158127
*Eurymeloefernandezi*	Spain: Islas Canarias: La Palma, Los Sauces	**mel 07048**	MW158267	MW158069	MW157973	MW158128
*Eurymeloeganglbaueri*	Italy: Lazio: Tarquinia	**mel 81064**	MW158268	MW158070	MW157974	MW158129
*Eurymeloeganglbaueri*	Italy: Lazio: Tarquinia	**mel 81065**	MW158269	MW158071	MW157975	MW158130
*Eurymeloeganglbaueri*	Italy: Sardinia: 4 km North-West of Orgosolo	**ASV19011**		OM918705		OM925566
*Eurymeloeglazunovi*	Romania: Dobruja: Istria	**mel 07001**	MW158265	MW158067	MW157971	MW158126
*Eurymeloegomari*	Morocco: 9 km South-West of Moulay Abdeselam	**ASV18019**	OM936883	OM918704		OM925565
*Eurymeloegomari*	Morocco: Tangier-Tetouan: Chaouen, Talassemtane National Park	**mel 81063**	MW158275	MW158076	MW157981	MW158136
*Eurymeloeibericus*	Spain: Ávila: Villanueva del Campillo, Puerto de Villatoro	**mel 06039**	MW158307	MW158090	MW157989	MW158144
*Eurymeloeibericus*	Spain: Ávila: Hoyos del Espino, Plataforma de Gredos	**mel 81039**	MW158308	MW158091	MW157990	MW158145
*Eurymeloemediterraneus*	Spain: Cádiz: Puerto Real	**mel 04255**	MW158221	MW158049	MW157967	MW158122
*Eurymeloemediterraneus*	Morocco: Moulay Abdelsalam	**mel 07010**	MW158222	MW158050	MW157968	MW158123
*Eurymeloemediterraneus*	Spain: Cuenca: Saelices	**mel 07147**	MW158224	MW158052	MW157970	MW158125
*Eurymeloemediterraneus*	Morocco: Tetouan: Agnan, Sierra del Haus	**mel 81066**	MW158223	MW158051	MW157969	MW158124
*Eurymeloemurinus*	Morocco: Marrakesh-Safi: 2 km North of Aguelmouse, Tizi n’Tichka, High Atlas	**mel 81018**	MW158270	MW158072	MW157976	MW158131
*Eurymeloemurinus*	Spain: Madrid: Colmenar Viejo	**mel 81053**	MW158271		MW157977	MW158132
*Eurymeloenanus*	Spain: Madrid: 7 km South of Tielmes	**mel 01028**	MW158273	MW158074	MW157979	MW158134
*Eurymeloenanus*	Spain: Madrid: Tielmes	**mel 81042**	MW158274	MW158075	MW157980	MW158135
*Eurymeloenanus*	Spain: Toledo: Villacañas, Sierra del Romeral	**mel 05001**	MW158272	MW158073	MW157978	MW158133
*Eurymeloeorobates* sp. nov.	Spain: Guadalajara: Puerto de la Quesera	**ASV18002**	OM936884		OM925567	
*Eurymeloeorobates* sp. nov.	Spain: Guadalajara: Puerto de la Quesera	**ASV18003**	OM936885		OM925568	
*Eurymeloerugosus*	Germany: Saxony-Anhalt	**ZFMK-TIS-2003300**	KU918912.1			
*Eurymeloetuccia*	Spain: Menorca: 2 km South of Binimella	**mel 06034**	MW158276	MW158077	MW157982	MW158137
*Eurymeloetuccia*	Spain: Islas canarias: La Palma: Don Pedro	**mel 07058**	MW158277	MW158078	MW157983	MW158138
*Eurymeloetuccia*	Spain: Almería: La Mela	**mel 81001**	MW158278	MW158079	MW157984	MW158139
*Eurymeloetuccia*	Portugal: Algarve: Sagres, Praia do Martinhal	**mel 81002**	MW158279	MW158080	MW157985	MW158140
*Eurymeloetuccia*	Morocco: Larache: Lixus	**mel 81006**	MW158280	MW158081	MW157986	MW158141
*Lampromeloeaff.variegatus*	Morocco: Marrakesh-Safi: 5.5 km North-East of Aguelmouse, Tizi n’Tichka, High Atlas	**mel 81010**	MW158202	MW158033	MW157953	MW158108
*Lampromeloecavensis*	Morocco: Casablanca-Settat: Ouled Bahmad	**mel 06011**	MW158201	MW158032	MW157952	MW158107
*Lampromeloevariegatus*	Spain: Salamanca: 5 km West of Palencia de Negrilla	**mel 05015**	MW158203	MW158034	MW157954	MW158109
*Lampromeloevariegatus*	Hungary: Komárom-Esztergom: Vèrtesszölös	**mel 81068**	MW158204	MW158035	MW157955	MW158110
Meloe (Anchomeloe) autumnalis	Spain: Guadalajara: Villanueva de la Torre	**mel 04246**	MW158189	MW158025	MW157949	MW158104
Meloe (Anchomeloe) autumnalis	Spain: Zaragoza: El Frago	**mel 10070a**	MW158191	MW158027	MW157951	MW158106
Meloe (Anchomeloe) autumnalis	Morocco: Tangier-Tetouan: Chaouen, Djebel Tissouka	**mel 81071**	MW158190	MW158026	MW157950	MW158105
Meloe (Meloe) proscarabaeus	Spain: Menorca: 3.5 km South-West of Fornells	**mel 06026**	MW158148	MW157993	MW157939	MW158094
Meloe (Meloe) proscarabaeus	Morocco: Souss-Massa: 2 km South of Chafarni	**mel 81007**	MW158150	MW157995	MW157941	MW158096
Meloe (Meloe) proscarabaeus	Hungary: Tolna: Bátaapáti	**mel 06004**	MW158151	MW157996	MW157942	MW158097
Meloe (Meloe) proscarabaeus	Italy: Tuscany: Alberese	**mel 81082**	MW158149	MW157994	MW157940	MW158095
Meloe (Meloe) tropicus	Guatemala: El Quiché: 9 km North-East of Santa Cruz Quiché	**mel 81075**	MW158188	MW158024	MW157948	MW158103
Meloe (Meloe) cf. violaceus	Hungary: Tolna: Mőcsény	**mel 07033**	MW158186	MW158022	MW157946	MW158101
Meloe (Meloe) cf. violaceus	Hungary: Vas: Csákánydoroszló	**mel 07036**	MW158187	MW158023	MW157947	MW158102
Meloe (Meloe) violaceus	Spain: Ávila: Hoyos del Espino	**mel 05024**	MW158183	MW158019	MW157943	MW158098
Meloe (Meloe) violaceus	Spain: León: Correcillas: Pico Polvareda	**mel 81051**	MW158185	MW158021	MW157945	MW158100
Meloe (Meloe) violaceus	Spain: León: Correcillas: Pico Polvareda	**mel 81052**	MW158184	MW158020	MW157944	MW158099
*Meloegoniuscicatricosus*	Hungary: Pest: Tatárszentzgyörgy	**mel 06002**	MW158208	MW158038	MW157959	MW158114
*Meloegoniuscicatricosus*	Hungary: Komárom-Esztergom: Vèrtesszölös	**mel 81069**	MW158209	MW158039	MW157960	MW158115
*Mesomeloecoelatus*	Morocco: Guelmine-Smara: Reg Labyad	**Mcoelatus_labyad**	MW805179			
*Mesomeloecoelatus*	Morocco: Guelmine-Smara: Jbel Ouarkziz	**Mcoelatus_ouarkziz**	MW805180			
*Mesomeloeottomerkli*	Qatar: 1.8 km West of Al Marrawnah	**mel Qatar1**	HG003653	MW158044	MW157962	MW158117
*Mesomeloeottomerkli*	Qatar: 1.8 km West of Al Marrawnah	**mel Qatar2**	HG003654	MW158045	MW157963	MW158118
*Physomeloecorallifer*	Spain: Madrid: 5 km South-East of Agustín de Guadalix, 618 m asl	**mel 09051**	MW158210	MW158040	MW157961	MW158116
*Taphromeloeerythrocnemus*	Morocco: 2.5 km North-West of Douar Azerzou	**ASV18007**	MW158309	MW158092	MW157991	MW158146
*Treiodousgracilicornis*	Guatemala: El Quiché: 3.4 km North of Uspantán	**mel 81077**	MW158205	MW158036	MW157956	MW158111
*Treiodousgracilicornis*	Mexico: Guerrero: 5 km West of Carrizal de Bravo	**mel 874**	MW158206		MW157957	MW158112
*Treiodouslaevis*	Mexico: Guanajuato: Dolores Hidalgo	**mel 08094**	MW158207	MW158037	MW157958	MW158113
*Lyttavessicatoria*	Spain: Ourense: A Acea	**mel 05073**	MW158147	MW157992		MW158093
*Phodagaalticeps*	Mexico: Baja California Norte: Ejido Luchadores del Desierto, Northwest of Laguna Salada	**KRN14**	MK024506	MK024642		MK024601
*Cordylospastafulleri*	USA: California: 4.8 km North-East of Big Pine	**KRN23**	MK024478	MK024619		MK024572

The molecular data set consisted of two mitochondrial (COI and 16S rRNA) and two nuclear (Wg and 18S rRNA) gene fragments from 66 specimens (including the four new ones). All sequences were compiled and revised using Sequencer v. 4.9 and aligned with MAFFT (Katoh and Toh 2008). Final alignments were visually inspected with Mesquite v. 3.04 (Maddison and Maddison 2019). Phylogenetic analyses using a Bayesian inference approach, as implemented in MrBayes v. 3.2.3 (Ronquist et al. 2012), were performed on a combined data set consisting of 2917 bp from the four mitochondrial and nuclear sequences (COI, 16S, Wg, 18S) (Table 1). Analyses, which started with a randomly generated tree, consisted of four Metropolis-coupled Markov chains Monte Carlo (one cold, three heated) and two simultaneous runs of 10 × 10^6^ generations each, sampling every 1000 generations. We discarded the first 25% of the obtained trees as burn-in and generated the consensus tree in MrBayes. Posterior clade probabilities were used to assess nodal support.

To delimit species, we adopted the evolutionary species concept (Wiley 1978; Wiley and Mayden 2000) in which a species is considered “a single lineage of ancestral descendant populations of organisms which maintains its identity from other such lineages and which has its own evolutionary tendencies and historical fate” (Wiley 1978: 18). This concept combines implications derived from the phylogenetic species concept, such as reciprocal monophyly, with additional subjective properties (e.g., phenetic distinguishability and reproductive isolation, among other lines of evidence) that can be used to assess the historical fate of lineages (Ruiz and García-París 2015; Sánchez-Vialas et al. 2020).

## ﻿Results

### ﻿Phylogenetic relationships within *Eurymeloe*

**Figure 1.**
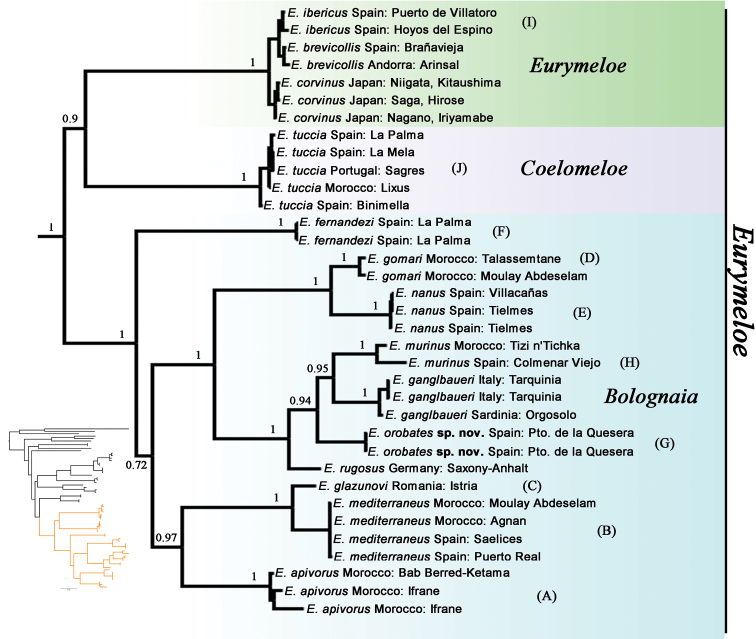
Bayesian phylogeny of the genus *Eurymeloe* based on the concatenated matrix of mitochondrial and nuclear genes (COI, 16S, Wg, 18S). The three subgeneric taxonomic categories are indicated as follows: *Eurymeloe* (in green), *Coelomeloe* (in violet) and *Bolognaia* (in blue). Numbers on the nodes represent the posterior probabilities of the clades. Letters in parentheses (**A–J**) correspond to the species portrayed in Fig. 2. Phylogeny in lower left shows the outgroups (black) and the lineage of *Eurymeloe* (orange). Additional information for each specimen is provided in Table 1.

Our Bayesian phylogenetic tree, which has a topology similar to the one presented by Sánchez-Vialas et al. (2021), recovered three major lineages within the genus *Eurymeloe* (Fig. 1). The first lineage is represented by the type species of the genus *Eurymeloe*, *E.brevicollis* (Panzer, 1793), plus the species *E.ibericus* (Reitter, 1895) and *E.corvinus* (Marseul, 1877), which together form a clade with *E.tuccia* (Rossi, 1790), the type species of the subgenus Coelomeloe. These two clades together form the sister group of the clade comprising the remaining species of *Eurymeloe* that were previously included in the *E.rugosus* species group (sensuBologna 1988).

With respect to the internal taxonomic structure of *Eurymeloe* (sensuSánchez-Vialas et al. 2021), it consists of three main clades, all morphologically diagnosable, with the following subgeneric status: (1) *Coelomeloe*; (2) *Eurymeloesensu stricto* (s. str.), which includes the species in Bologna’s (1988)*E.brevicollis* species group, and (3) the lineage including all species within the *E.rugosus* species group (Fig. 1). As the name-bearing type species of *Eurymeloe* (*E.brevicollis*) lies within the *E.brevicollis* species group, *Eurymeloe* (s. str.), the clade formed by the *E.rugosus* species group requires a new subgeneric name (as there is no name available for this group). We propose and describe herein a new subgenus of *Eurymeloe* to accommodate the species of Bologna’s *M.rugosus* species group (sensuBologna 1988): Bolognaia subgen. nov. We also redefine the subgenus Eurymeloe.

**Figure 2.**
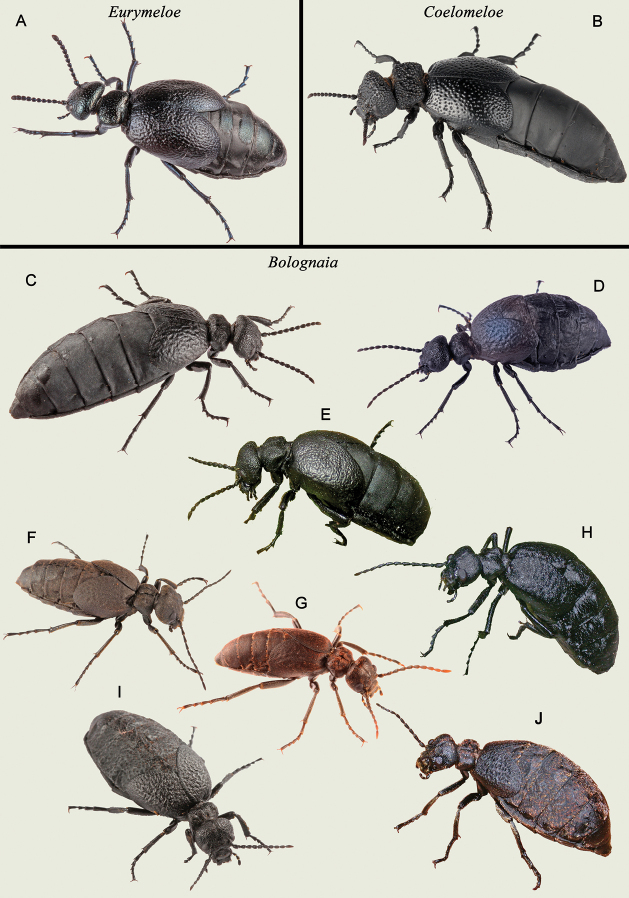
Habitus of some living adult specimens of the current subgenera of *Eurymeloe***A** subgenus Eurymeloe, represented by a specimen of Eurymeloe (Eurymeloe) ibericus from Puebla de la Sierra, northern Madrid, Spain **B** subgenus Coelomeloe, represented by a specimen of Eurymeloe (Coelomeloe) tuccia found 3 km east of Celín, Sierra de Gádor, Almería, Spain **C–I** subgenus Bolognaia**C**Eurymeloe (Bolognaia) affinis from the surroundings of Bab Taza, Rif Mountains, Morocco **D**E. (Bolognaia) mediterraneus (type species of the subgenus Bolognaia) from Puebla de la Sierra, northern Madrid, Spain **E**E. (Bolognaia) glazunovi from Istria, Romania **F**E. (Bolognaia) gomari from the surroundings of Bab Taza, Rif Mountains, Morocco **G**E. (Bolognaia) nanus from Alcázar de San Juan, Ciudad Real, Spain **H**E. (Bolognaia) fernandezi from Los Sauces, La Palma, Canary Islands **I***Eurymeloeorobates* sp. nov., from Puerto de la Quesera, Guadalajara, Spain **J**Eurymeloe (Bolognaia) murinus from Tizi n`Tichka, High Atlas, Morocco. Photographs: **A–C, F, G, I** (ASV) **E, H, J** (MGP) **D** (J. Aznar González de Rueda).

*Bolognaia* is comprised of three main lineages: one represented by *E.fernandezi*, an endemic of the Canary Islands, which resolved as the sister taxon to the other two lineages formed, respectively, by *E.mediterraneus* (Müller, 1925) and its closely related species and by *E.murinus* (Brandt and Erichson 1832) and its related species, including the new species of *Eurymeloe* from central Spain. The sequences of the new samples from Spain resolved as a distinctive lineage that is the sister taxon to the clade formed by *E.murinus* and *E.ganglbaueri*. This new lineage is not morphologically congruent with any other described species of *Eurymeloe* (Fig. 2).

Lastly, our molecular phylogenetic analysis confirmed the species identification of the other two newly sequenced specimens as *E.gomari* and *E.ganglbaueri*, and their relationship with the other specimens. As a result, the northern limit of the distribution range of the Moroccan endemic *E.gomari*, which was previously known only from its type locality, has been expanded by approximately 37 km to the northwest along the Rif Mountains. The new specimen of *E.ganglbaueri* from Sardinia resolved, as expected, within the same clade as the conspecific Italian continental samples.

### ﻿Redefinition of the genus *Eurymeloe*

Given the new systematic arrangement proposed here for *Eurymeloe* as three subgenera (*Eurymeloe*, *Coelomeloe*, and Bolognaia subgen. nov.), we consider it necessary to present a revised diagnosis of *Eurymeloe* based on the morphology of adult specimens.

#### ﻿Genus Eurymeloe

Taxon classificationAnimaliaColeopteraMeloidae

Reitter, 1911

4CDB0303-7A76-5ED6-A343-F425C1AE114C

##### Type species.

*Meloebrevicollis* Panzer, 1793, by subsequent designation of Pinto and Selander (1970).

##### Description

**(adult).** Size small to medium (6–36 mm), with diverse appearance, ranging from very robust to comparatively slender. Body integument colour variable, black, dull grey or dark brown (exceptionally sandy brown) to moderately metallic blue, opaque, bright, silky or sometimes with an oily shininess (Fig. 2A). Body pubescence short, sometimes quite distinct (*Bolognaia*) or very short, recumbent, often almost imperceptible (subgenus Eurymeloe) or absent dorsally (*Coelomeloe*; Fig. 2B), variable in colour, from yellowish to reddish brown and black. Head rounded, sides straight to arched, convergent to eyes. Eyes small or medium, usually subreniform, variably protruding, without longitudinal depressions behind them. Antennae unmodified in males, moniliform or submoniliform, robust or slender, short to moderate length, not reaching posterior margin of pronotum (*Eurymeloe*, *Coelomeloe*) or often reaching or even surpassing it (*Bolognaia*). Antennomeres subcylindrical or subconical, relatively robust or slender, highly variable width/length ratio, V to VII neither enlarged nor geniculated. Clypeus transverse, approximately twice as wide as long. Frontoclypeal suture angulated. Labrum wide, fore margin broadly emarginate. Maxillary and labial palpomeres unmodified. Mandibles robust, regularly and strongly curved on the outer margins. Pronotum from flat (*Coelomeloe*) to moderately convex (*Eurymeloe*, *Bolognaia*), subrectangular (*Coelomeloe*, *Eurymeloe*), subhexagonal or trapezoidal (*Bolognaia*), transverse or very transverse, usually equal to or more than 1.5 × as wide as long (exceptionally, 1.2–1.3 × as wide as long), with sides subparallel or converging backward; posterior margin usually broadly emarginated, with base not incised in the middle. Head and pronotum punctation from fine and scattered, sometimes almost absent (*Eurymeloe*), to somewhat deep and dense (*Bolognaia*), even very broad, dense, and deep, foveate in appearance (*Coelomeloe*). Hind margin of mesonotum straight or weakly arcuate. Metanotum short and barely visible, covered by the base of the elytra. Mesosternum short and wide, lacking scutum. Mesepisterna meet or not at the midline of the body. Elytra short and dehiscent, imbricate basally, not completely covering abdomen, smooth to densely coriaceous, rugose (*Eurymeloe*, *Bolognaia*) or with a surface densely foveate (*Coelomeloe*). Hind wings absent. Legs normal, unmodified in male, robust or more or less slender, pilose. Tibiae with two spurs at apex; outer spur of metatibiae widened and obliquely truncate, spoon-shaped. Tarsomeres with or without hair pads or dense setose pubescence on the inferior sides. Tarsal claws smooth, with distinct lower blades. Abdomen large, inflated, hypertrophied. Abdominal terga with medium or small highly sclerotised central plates. Last abdominal ventrite broadly emarginated at hind margin in males. Male genitalia: gonoforceps evenly sclerotised with gonostyli from moderately short to elongate, with distal regions more or less wide, usually digitiform (in lateral view), rounded at apex; gonocoxal plate broadly widened at the middle (in dorsal view); aedeagus robust (*Eurymeloe*, *Coelomeloe*) or relatively slender (*Bolognaia*), usually shorter than or approximately equal in length to the gonoforceps (in some species of *Bolognaia*, sometimes a little longer than the gonoforceps), with two dorsal hooks and one endophallic hook.

**Larva.** The morphological traits of previously known first instar larvae of *Eurymeloe* (triungulines), including *Coelomeloe*, and the descriptions of the triungulines of several additional species of *Eurymeloe*, have been synthesised and studied by Di Giulio et al. (2013, 2014). We herein refer to these works for larval morphological traits.

##### Taxonomic remarks.

The adult instar of species of the genus *Eurymeloe* is morphologically diverse, and the three subgenera can be recognised based on this diversity. Adults of the subgenera *Eurymeloe*, *Coelomeloe*, and the newly described *Bolognaia* are distinguishable particularly by the shape of the antennae and the pronotum, the macrosculpture of the pronotum, body integument and pilosity, and the punctation of the head, pronotum, and elytra, among other traits.

### ﻿Description of the subgenus

#### ﻿ Bolognaia

Taxon classificationAnimaliaColeopteraMeloidae

Ruiz, García-París, Sánchez-Vialas & Recuero, subgen. nov.

08071F98-8C5D-549A-84C8-4588186ABE88

https://zoobank.org/6B062E01-EF30-47F3-8ED2-42394FE7D532

##### Type species.

*Meloemediterraneus* Müller, 1925, by present designation.

##### Description (adult).

Size small or medium to large (8–36 mm). Body integument black, dull grey or dark brown, occasionally sandy brown [*E.pallidicolor* (Martínez de la Escalera, 1909)], with an opaque, silky or bright appearance, never bluish or metallic (Fig. 2C–J). Body pubescence quite distinct, black, yellowish, whitish or golden, short or very short. Head rounded, temples usually forming a regular arc, except in *E.murinus* (Brandt and Erichson 1832) and *E.affinis* (Lucas, 1847), which have strongly enlarged temples; occiput usually weakly concave. Medium-sized eyes [smaller in *E.affinis* and *E.apivorus* (Reitter, 1895)], subreniform, moderately protruding, without a longitudinal depression behind them. Antennae moniliform, normally slender, not thickened towards the apex; long or medium in length, usually reaching the posterior margin of the pronotum or exceeding it; unmodified in males, straight. Antennomeres IV–IX subcylindrical, always longer than wide. Clypeus transverse, approximately twice as wide as long. Labrum wide, fore margin broadly emarginate. Mandibles robust, regularly curved along the outer margins. Pronotum slightly convex, transverse, mainly subhexagonal or subtrapezoidal, wider than long, usually 1.4–2.1 × as wide as long [in *E.fernandezi*, 1.2–1.3 × as wide as long], sides not parallel, converging backward, posterior margin broadly emarginated, posterior corners rounded. Pronotum surface variable, with or without a depressed area or groove in the middle, frequently with two depressed or smooth areas, diffuse, on both sides of the disc. Head and pronotum punctation fine to coarse, of variable density, always with pubescence. Posterior margin of mesonotum straight or weakly arcuate. Mesepisterna usually not meeting at the midline of the body. Elytra short and dehiscent, smooth to strongly rough, usually rugulose. Legs normal, usually slender, pubescent. Tarsomeres without hair pads on the inferior side, though some species [e.g., *E.nanus* (Lucas, 1847), *E.baudueri* (Grenier, 1863), *E.gomari*] have fairly dense setose pubescence that appears as small and short brushes. Last abdominal ventrite broadly and deeply emarginated at the hind margin in males. Male genitalia: Gonostyli usually elongate, distal regions narrow with their apices acuminate or digitiform in lateral view; gonocoxal plate long, usually narrow and slightly widened at the middle in dorsal view; aedeagus usually elongate, equally long as or longer than gonoforceps.

##### Etymology.

The name *Bolognaia*, formed by the noun “Bologna” plus the Italian suffix “–aia” derived from the Latin “–aria” (used, in this case, to form a word meaning an animal associated with the specified noun Bologna), is in honour of Marco A. Bologna, a distinguished Italian entomologist specialising in the Meloidae and a friend who, among other excellent works, was able to clarify, for the first time, the complex taxonomy of the small-sized species of *Eurymeloe* related to *E.rugosus* for which the new subgenus is here erected.

##### Taxonomic remarks.

We selected *M.mediterraneus* as the type species of *Bolognaia* because it is a morphologically well-characterised species, with low morphological or genetic intraspecific geographic differentiation (Bologna 1988, 1991; Sánchez-Vialas et al. 2021), and without nomenclatural or identity problems associated to synonyms (García-París et al. 2010; Bologna 2020a). *Bolognaia*, a monophyletic subgenus, largely corresponds to the *E.rugosus* species group of *Eurymeloe* defined by Bologna (1988, 1991). It includes species whose adults are characterised mainly by the following traits: small or medium body size; black, dull grey, or dark brown body colour; a distinctive black or pale-coloured (yellowish, whitish, or golden) pilosity; elongated and subcylindrical antennomeres that are longer than wide; and generally marked punctation and rugosity.

Based on molecular data (this work) and adult morphology (see Reitter 1895; Martínez de la Escalera 1909; Pliginskij 1910; Pardo Alcaide 1951; Kaszab 1958, 1983; Bologna 1988, 1991, 1994a, 1994b; Ruiz and García-París 2009, 2015; García-París and Ruiz 2011; Di Giulio et al. 2013), we include within the subgenus Bolognaia the following species: Eurymeloe (Bolognaia) affinis (Lucas, 1847), E. (B.) apivorus (Reitter, 1895), E. (B.) apenninicus (Bologna, 1988), E. (B.) baamarani (Ruiz and García-París 2015), E. (B.) baudii (Leoni, 1907), E. (B.) baudueri (Grenier, 1863), E. (B.) fernandezi, E. (B.) flavicomus (Wollaston, 1854), E. (B.) ganglbaueri, E. (B.) glazunovi (Pliginskij, 1910), E. (B.) gomari, E. (B.) kandaharicus (Kaszab, 1958), E. (B.) mediterraneus, E. (B.) murinus, E. (B.) nanus, E. (B.) omanicus (Kaszab, 1983), E. (B.) pallidicolor, and E. (B.) rugosus.

According to our molecular analyses (Fig. 1), three sublineages can be recognised within *Bolognaia*: two generally corresponding to Bologna’s (1988) subgroups A and B, defined by presenting, respectively, an entirely black body pilosity (in our analyses, *E.mediterraneus*, *E.apivorus*, and *E.glazunovi*) or a pale-coloured (whitish, yellowish, reddish yellow, or golden) pilosity over the entire body or parts of it [in our analyses, *E.ganglbaueri*, *E.murinus*, *E.nanus*, *E.gomari*, *E.rugosus*, and *Eurymeloeorobates* sp. nov. from central Spain]. Notably, based on our molecular analyses, *E.rugosus*, which was included in Bologna’s (1988) subgroup A, appears to be genetically more related to the species included in his subgroup B. In this regard, following a detailed examination of some specimens belonging to *E.rugosus*, we observed that several have inconspicuous brownish and reddish to yellowish setae (but not tufts) on their abdominal tergites, similar to those observed on the morphologically related species *E.apenninicus* (JLR, pers. obs.). In fact, Escherich (1890) and Bologna (1988, 1991) pointed out that some specimens of *E.rugosus* show yellowish brown setae on the last abdominal tergites; these correspond to the named var. abdominalis (Escherich 1890) (which has even been confused with *E.ganglbaueri*, see Bologna 1988: 247). Likewise, *E.ganglbaueri*, which presents a golden-yellow pilosity on a part of the body, resolved as genetically more related to species in subgroup B. The third sublineage diverged from its sister group, the A and B sublineages, during the Middle Miocene (Sánchez-Vialas et al. 2021). This sublineage is composed of only one species, *E.fernandezi*, an endemic of the Canary Islands that is morphologically singular and isolated within the subgenus (Pardo Alcaide 1951; Bologna 1988, 1991, 1994a; Ruiz and García-París 2015).

For practical purposes, but also supported by our analyses and some morphological traits (mainly, pilosity colour, integument aspect, pronotum punctation, and elytra rugosity; see Bologna 1988; Ruiz and García-París 2009, 2015), we redefine the specific composition of the subgroups established by Bologna (1988) within *Bolognaia* (defined as the *E.rugosus* species group by Bologna 1988) as follows:

(1) group A or *E.mediterraneus* group (now renamed), characterised mainly by a dark body pilosity (black or dark brown) and a black body integument that is usually glossy, semi-glossy, or silky in appearance [exceptionally, it is matte as in E. (B.) baamarani]. This group integrates the following species: E. (B.) affinis, E. (B.) apivorus, E. (B.) baamarani, E. (B.) baudii, E. (B.) glazunovi, and E. (B.) mediterraneus. In some specimens of E. (B.) mediterraneus, particularly those from Sardinia (Bologna 1988, 1991), the pilosity of the temples, pronotum and, sometimes, abdomen, is brown. The unstudied E. (B.) affinis setosus Escherich, 1890 from Algeria, which differs from the typical form of the species by the presence of isolated yellowish setae along the abdominal tergites, among other traits (e.g., smaller size, constant frontal furrow, and different elytral sculpture) (Escherich 1890; Bologna 1988, 1991; Di Giulio et al. 2013), possibly constitutes a distinct species, as mentioned by Di Giulio et al. (2013) and previously suggested by Escherich (1890) and Peyerimhoff (in Cros 1934: 90).

(2) group B or *E.murinus* group, characterised mainly by a pale-coloured (reddish, golden, brownish, yellowish, or whitish) body pilosity, either over the entire body or parts of it, and a body integument that is usually greyish, greyish black, or dark brown and opaque; exceptionally, it is glossy black as in E. (B.) apenninicus and E. (B.) rugosus. This group comprises the following species: E. (B.) apenninicus, E. (B.) baudueri, E. (B.) flavicomus, E. (B.) ganglbaueri, E. (B.) gomari, E. (B.) kandaharicus, E. (B.) murinus, E. (B.) nanus, E. (B.) omanicus, E. (B.) pallidicolor, and E. (B.) rugosus. Within this group, E. (B.) apenninicus and E. (B.) rugosus can be clearly differentiated from the others by having a glossy black body integument and dark reddish brown (sometimes almost black) body setation, with scattered and sparse yellowish brown short setae on the abdominal tergites that are often barely noticeable.

(3) group C, composed by only E. (B.) fernandezi, well characterised morphologically within *Bolognaia* (see Pardo Alcaide 1951; Ruiz and García-París 2015).

Bologna (1988, 1990) integrated *M.saharensis* (= *M.otini* Peyerimhoff, 1949, *M.marianii* Kaszab, 1983; see Ruiz et al. 2010; Bologna 2020a) and *M.vignai* in the *E.rugosus* species group. Ruiz et al. (2010) considered the closely related *E.saharensis* and *E.vignai* as morphologically isolated and proposed a new group for them. As neither *E.saharensis* nor *E.vignai* have been studied at the molecular level, we have tentatively ascribed them to *Bolognaia* as Eurymeloe (Bolognaia) saharensis (Chobaut, 1898) and *E. (B.) vignai* (Bologna, 1990).

Sánchez-Vialas et al. (2021) considered the six Asian species that Bologna (1988) included in the *E.rugosus* species group as belonging to *Eurymeloe*. These species are *Eurymeloeheptapotamicus* (Pliginski, 1910), *E.primaeveris* (Kaszab, 1958), *E.punjabensis* (Kaszab, 1958), *E.schmidi* (Kaszab, 1978), *E.scutellatus* (Reitter, 1895), and *E.subsetosus* (Reitter, 1895). However, the available information on these taxa is currently insufficient to assign them to the subgenus Bolognaia; therefore, they require further study at both the morphological and the molecular levels.

### ﻿Redefinition of the subgenus

#### ﻿ Eurymeloe

Taxon classificationAnimaliaColeopteraMeloidae

Reitter, 1911

CC6F6F27-FA23-5FD2-87AB-6AC80A0CF1D6

##### Type species.

*Meloebrevicollis* Panzer, 1793 (by subsequent designation of Pinto and Selander, 1970).

##### Description (adult).

Size small or medium (6–30 mm), usually robust in appearance. Body integument colour black to moderately metallic blue, bright, silky, or with an oily shininess (Fig. 2I). Body pubescence very short, recumbent, or absent on the head and pronotum. Head rounded, sides almost straight, convergent to the eyes. Eyes small, subreniform, weakly protruding, and without longitudinal depression behind them. Antennae submoniliform, robust, short or medium in length, usually not reaching the posterior margin of the pronotum, smoothly thickened towards the apex in some species (e.g., *E.brevicollis*); in males, unmodified. Antennomeres subcylindrical or subconical, V to VII (in some species IV to IX, e.g., *E.brevicollis*), wider than long or, at most, as wide as long. Clypeus transverse, approximately twice as wide as long. Labrum wide, fore margin broadly emarginate. Mandibles robust, often curved along the outer margin. Pronotum slightly or moderately convex, very transverse, usually more than 1.7 × wider than long, sides not parallel and obtusely rounded, posterior margin broadly emarginated, posterior corners rounded. Pronotum surface slightly variable, moderately convex, usually with a weak, diffuse, median longitudinal groove. Head and pronotum punctation from fine and scattered, sometimes almost absent, to deep and dense, with or without (*E.brevicollis*) very short pubescence. Hind margin of mesonotum straight or weakly arcuate. Elytra short and dehiscent, smooth to densely coriaceous or rugose. Legs normal, robust, pilose. Tarsomeres without hair pads or dense setose pubescence on the inferior side. Last abdominal ventrite broadly emarginated in males. Male genitalia: Gonostyli moderately short, distal regions wide, usually digitiform in lateral view, rounded at apex; gonocoxal plate broadly widened at the middle in dorsal view; aedeagus robust, relatively shorter than the gonoforceps or, at most, similar in length.

##### Taxonomic remarks.

According to the present definition of the subgenus Eurymeloe, it is correlated with the *E.brevicollis* species group defined by Bologna (1988). It comprises a heterogeneous group of species characterised mainly by the following features in adults: small or medium in size, with a robust appearance; metallic blue or black body colour; reduced pilosity that is very scarce and short, often almost absent; wide antennomeres with V–VII usually wider than long; and variable head and pronotum punctation and elytral rugosity (see Bologna 1988, 1991).

Bologna (1988) tentatively included 22 species in the *E.brevicollis* species group. However, as this author pointed out, most of these species are very poorly known and, in some cases, the only morphological information on them is from the original description. As a result, the internal taxonomy of *Eurymeloe* s. str. is very complex and unclear (Bologna 1988).

On the basis of the molecular and morphological data (Reitter 1895, 1911; Escherich 1896; Martínez de la Escalera 1914; Peyerimhoff 1926; Bologna 1988, 1991, 1994a, 1994b; García-París et al. 2010; Di Giulio et al. 2013; this study), we ascribe to *Eurymeloe* s. str. the following species: Eurymeloe (Eurymeloe) algiricus (Escherich, 1890) (or *E.brevicollisalgiricus*, see Bologna 2008), E. (E.) austrinus (Wollaston, 1854), E. (E.) brevicollis, E. (E.) corvinus (possibly co-specific with the previous species according to Di Giulio et al. 2013), E. (E.) crosi (Peyerimhoff, 1926), E. (E.) curticornis (Martínez de la Escalera, 1914) (or *E.brevicolliscurticornis*, see Bologna 2008, 2020a), E. (E.) ibericus, and E. (E.) lederi (Reitter, 1895). The taxonomic positions of *E.luctuosus* (Brandt & Erichson, 1832) (related to *E.crosi*) and *E.scabriusculus* (Brandt & Erichson, 1832) (morphologically similar to *E.baudii* and *E.glazunovi*, both now included in *Bolognaia*) are still uncertain (Bologna 1988, 1991), and their assignment to *Eurymeloe* s. str. requires further studies.

Another 13 species [from Palaearctic Asia, except *E.aleuticus* (Borchmann, 1942), from the Aleutian Islands] were provisionally assigned by Bologna (1988) to the *E.brevicollis* species group: *Eurymeloealeuticus*, *E.curticollis* (Kraatz, 1882), *E.escherichi* (Reitter, 1889), *E.frontalis* (Reitter, 1905), *E.gaberti* (Reitter, 1907), *E.laevipennis* (Brandt & Erichson, 1832), *E.lobicollis* (Fairmaire, 1891), *E.mandli* (Borchmann, 1942), *E.mathiesseni* (Reitter, 1905), *E.primulus* (Semenow, 1903), *E.servulus* (Bates, 1879), *E.transversicollis* (Fairmaire, 1891), and *E.zolotarevi* (Pliginskij, 1914). As in the previous case, additional molecular and morphological studies are required to determine the subgeneric assignment of these species.

Regarding other species of *Eurymeloe*, Shapovalov (2012) described Meloe (Eurymeloe) sarmaticus Shapovalov, 2012 from Russia and Central Kazakhstan and considered it closely related to the Russian-Kazakh *E.aeneus* (Tauscher, 1812). The last species, together with *E.pusio* (Wellman, 1910) and *E.asperatus* (Tan, 1981), were considered *incertae sedis* by Bologna (1988). However, recently, Bologna (2020a) integrated them into *Eurymeloe* (at the subgenus level), although he still considers *E.aeneus* a doubtful ascription. We did not examine material of these species; therefore, we cannot add new information on their current taxonomic placement.

### ﻿Key to the subgenera of *Eurymeloe*

**Table:** 

1	Body entirely black and opaque. Body pubescence absent dorsally. Pronotum flat, subrectangular, transverse, depressed in middle of the base, with sides straight, parallel. Punctation of the head and pronotum very broad, dense, subcontiguous (less dense in Sicilian and southern Italian populations) and deep, clearly foveate in appearance (Fig. 2B). Elytral surface smooth, with punctation usually broad, dense and foveate (reduced and barely visible in Sicilian and southern Italian populations). Size medium to large (14–31 mm)	***Coelomeloe***
–	Body black, dull grey or dark brown (exceptionally sandy brown) to moderately metallic blue, bright, silky or more seldom opaque in appearance, sometimes with an oily shininess. Body pubescence quite distinct, or very short, recumbent, often almost imperceptible. Pronotum slightly to moderately convex, wider than long, with sides not parallel, more or less converging backward, and posterior angles usually broadly rounded. Head and pronotum punctation from fine and scattered, sometimes almost absent, to somewhat deep and dense, but never foveolate (Fig. 2A, C–J). Elytral surface smooth to densely coriaceous, subrugose or rugose, not foveolate. Size small to large (6–36 mm), but usually small to medium (6–22 mm)	**2**
2	Body colour black to moderately metallic blue, bright or silky. Overall appearance robust. Body pubescence very short, recumbent, almost imperceptible or even absent on the head and pronotum. Antennae compact, robust, sometimes smoothly thickened towards the apex, short or medium in length, not reaching the posterior margin of the pronotum. Antennomeres subcylindrical or subconical, V to VII (in some species IV to IX) wider than long or, at most, as wide as long	***Eurymeloe***
–	Body integument black, dull grey or dark brown, exceptionally sandy brown, with an opaque, silky or bright appearance, never bluish or metallic. Body pubescence quite distinct. Overall appearance more graceful, sometimes moderately robust. Antennae normally slender, not thickened towards the apex, long or medium in length, usually reaching the posterior margin of the pronotum or exceeding it. Antennomeres IV to IX subcylindrical, always longer than wide	***Bolognaia***

### ﻿Description of a new species of *Eurymeloe* from the Iberian Peninsula

Our molecular results revealed a distinctive lineage of *Eurymeloe* nested within the clade comprising *E.rugosus*, *E.murinus*, and *E.ganglbaueri*. This lineage, morphologically distinguishable from all its congeneric species, represents a new species that we herein describe.

#### ﻿ Eurymeloe (Bolognaia) orobates

Taxon classificationAnimaliaColeopteraMeloidae

sp. nov.

19D54712-AB88-5DAA-91C1-0B30E59F655A

https://zoobank.org/509BD098-E303-406E-AAE4-052059AC1865

##### Holotype.

**Figure 3.**
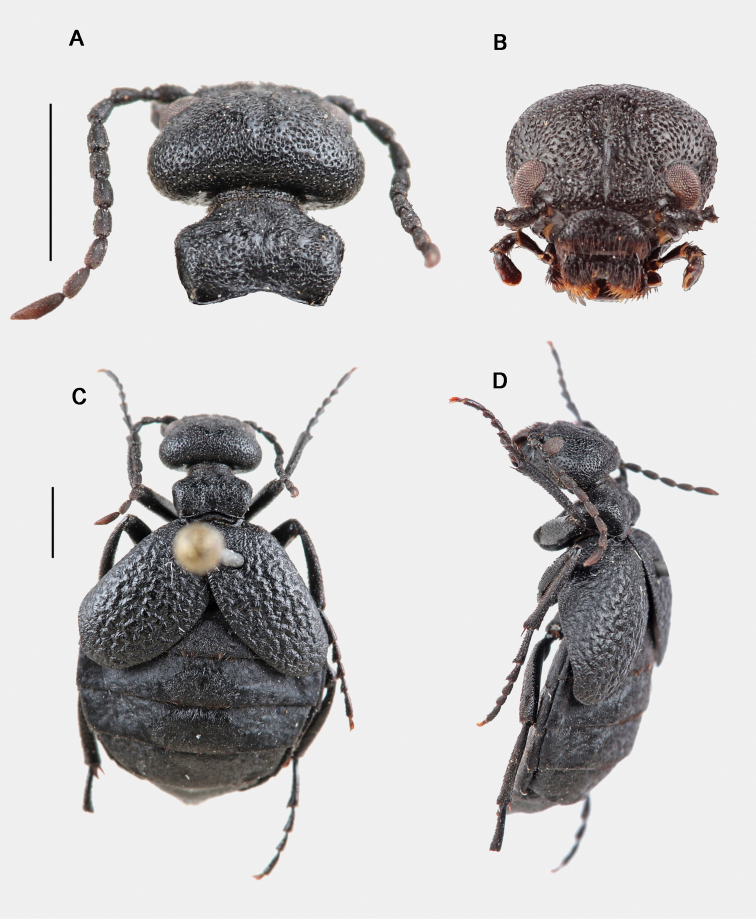
Holotype of *Eurymeloeorobates* sp. nov. MNCN 324740 **A** dorsal view of the head and pronotum **B** frontal view of the head **C** and **D** dorsal and dorsolateral views. Scale bars: 2 mm. Photographs: ASV.

**Figure 4.**
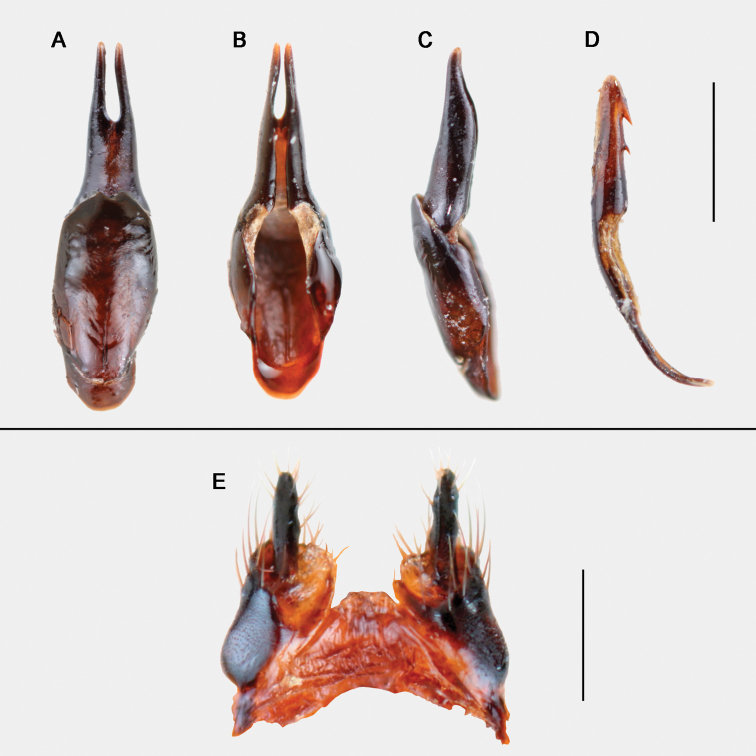
Genitalia of the male holotype MNCN 324740 (**A–D**) and of the female paratype MNCN 325409 (**E**) **A–C** ventral, dorsal and lateral views of the gonoforceps **D** lateral view of the aedeagus; scale bar: 1 mm **E** female gonostyli; scale bar: 0.2 mm. Photographs: ASV.

adult male (Fig. 3), labelled: “Puerto de la Quesera, Guadalajara, Spain, 41°12'32.2"N, 3°24'44.2"W, 1738 m, 15-XI-2015, F. Gutiérrez-Pérez et C. Cano leg.” [white label, printed]; “Holotypus Meloe (Bolognaia) orobates Sánchez-Vialas, Ruiz, Recuero, Gutiérrez-Pérez & García-París des. 2022” [white label, printed]; Holotipo [red label, printed]; MNCN_Ent 324740 [greyish label, printed]. Dissected and mounted genitalia (Fig. 4A–D). Dry-preserved, held at MNCN-CSIC.

##### Paratypes.

**Figure 5.**
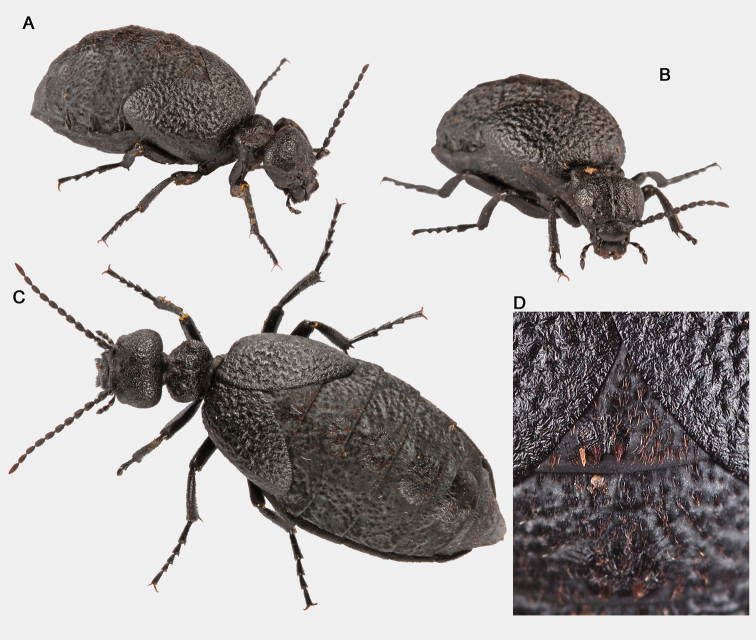
**A–C** habitus of a living female paratype (MNCN 325409) of *Eurymeloeorobates* sp. nov. **D** detail of the dorsal surface of the abdominal tergite I and the elytra. Note the brownish tufts. Photographs: ASV.

four adult females, labelled: two females: “Puerto de la Quesera, Guadalajara, Spain 41°11'30.11"N, 3°24'27.55"W, 1625 m, 22-V-2016, M. García París, A. Fernández Liger, A. Corral Lou leg. [white label, printed]; ASV 18002 and ASV 18003, respectively [white label, handwritten]; MNCN_Ent 325407 and MNCN_Ent 325408, respectively [white label, printed]. One adult female (Fig. 5): “Puerto de la Quesera, Guadalajara, Spain, 41°11'30.11"N, 3°24'27.55"W, 1625 m, 8-XII-2018, A. Sánchez-Vialas leg.” [white label, printed]; MNCN_Ent 325409 [white label, printed]. One adult female: “Puerto de la Quesera, Guadalajara, Spain, 41°12'58.10"N, 3°25'14.37"W, 1712 m, 28-XII-2021, A. Sánchez-Vialas leg.” [white label, printed]; MNCN_Ent 325410 [white label, printed]. –All paratypes labelled: “Paratypus, Meloe (Bolognaia) orobates Sánchez-Vialas, Ruiz, Recuero, Gutiérrez-Pérez & García-París des. 2022” [white labels, printed]. All paratypes are preserved in ethanol (except for the female gonostyli of the specimen MNCN_Ent 325409 [Fig. 4E], which was dissected, mounted on a piece of cardboard using DMHF, and preserved dry, bearing the following labels: “Puerto de la Quesera, Guadalajara, Spain, 41°11'30.11"N, 3°24'27.55"W, 1625 m, 8-XII-2018, A. Sánchez-Vialas leg.” [white label, printed]; “Paratypus, Meloe (Bolognaia) orobates Sánchez-Vialas, Ruiz, Recuero, Gutiérrez-Pérez & García-París des. 2022” [white label, printed]; Paratipo [red label, printed]), held at MNCN-CSIC.

##### Description of the holotype.

Total length (frons to apex of the tergite VIII): 11.05 mm. Length from the frons to the posterior margin of elytra: 6.55 mm. Maximum width (located slightly anterior to the apex of the elytra): 6.81 mm. Body relatively robust, with slender appendages (Fig. 3). Voluminous abdomen. Coloration black all over body and appendages, except tibial spines and tarsal claws, which are brownish. Integument finely microreticulated, silky or semi-glossy in appearance. Setation decumbent, reddish brown, fairly dark, sometimes almost black ventrally and on the legs, and very short on the head, pronotum and elytra, longer on the abdominal tergites, legs, pygidium and ventral region, and arranged in relatively conspicuous tufts or single reddish yellow setae on the sclerotised plates of the abdominal tergites.

Head voluminous, broadly rounded and clearly wider than the pronotum, weakly truncated on the posterior margin of the temples, with integument black, silky in appearance, finely microreticulated, and without longitudinal depressions behind the eyes (Fig. 3A, B). Maximum width in frontal view (at the level of the temples): 2.83 mm; minimum distance between the inner edges of the eyes: 1.83 mm; distance between the clypeus-frontal suture and the vertex (in frontal view): 1.81 mm. Temples wide and regularly rounded (Fig. 3B). Frons almost flat, with a weak and short longitudinal groove from the clypeus-frontal suture to the vertex, that is slightly deeper from the level of the eyes to the vertex; surface adjacent to the antennal insertions slightly elevated and with a weak and diffuse depression attached to the raised areas (Fig. 3B). Head punctation dense, consisting of rounded, markedly deep, closely positioned and subconfluent punctures, slightly larger in diameter in the frontal region and smaller towards the vertex, almost uniformly distributed, except on a narrow longitudinal mid-band on the frons, which is almost smooth (Fig. 3A, B). Head setation inconspicuous, short, decumbent, dark reddish brown, distributed according to the pattern of punctures in which it is inserted. Eyes medium-sized, subreniform and protruding, with upper lobes larger than the lower ones, barely notched at the level of the antennal insertions; clypeus-frontal suture deeply marked, weakly arcuate (Fig. 3B). Clypeus flat, transverse, subtrapezoidal, 2.1 × wider than long, with a brownish membranous anterior border; punctures medium-sized, separated by between 0.5 and 1 × their diameter, with the highest density on the sides; long setae homogeneously distributed, following the puncture pattern, directed forward, longest on the sides (Fig. 3B). Labrum-clypeus suture almost straight. Labrum transverse, 2.5 × wider than long, deeply emarginated in the middle, forming two clear lobes; punctures similar to those on the clypeus; setae longer on the lobes, following the punctation pattern, oriented forward and curved (Fig. 3B). Mandibles relatively robust, curved along the outer margins and notched in the distal region, glabrous at the apex, and scarcely pilose at the base. Maxillary and labial palps unmodified. Maxillary palps with palpomere I very short, subcylindrical (0.09 mm long, 0.1 mm wide); II longer, troncoconical, weakly curved in the proximal half (0.44 mm long, 0.21 mm wide); III troncoconical, shorter and wider than II (0.38 mm long, 0.23 mm wide); IV sub-trapezoidal, widened distally, broadly rounded at the apex and dorsoventrally flattened, with a narrow excavation along the distal margin (0.54 mm long, 0.3 mm wide); setae scattered and moderately long on palpomeres II and III, shorter and more scarce on palpomere IV. Labial palps short, not visible dorsally, with palpomere I subcylindrical, very short (0.11 mm long, 0.09 mm wide); II troncoconical (0.22 mm long, 0.15 mm wide); III similar in shape to the last maxillary palpomere (IV); setae as on maxillary palps.

Antennae with 11 antennomeres, moniliform, slender and long, surpassing the base of the pronotum when extended backward (Fig. 3A). Antennomeres not modified, subcylindrical or subconical, I–VIII black, semi-glossy, IX–XI dark brown, opaque but becoming reddish brown in XI. Antennomere I widened apically, subconical, ~ 1.92 × longer than wide (0.48 mm long, 0.25 mm wide); II short, subglobose, slightly wider than long (0.81 mm long, 0.82 mm wide); III–X subcylindrical, similar to each other, between 1.84 and 2.22 × longer than wide (III: 0.49 mm long, 0.22 mm wide; IV: 0.5 mm long, 0.24 mm wide; V: 0.48 mm long, 0.26 mm wide; VI: 0.46 mm long, 0.25 mm wide; VII: 0.49 mm long, 0.24 mm wide; VIII: 0.48 mm long, 0.23 mm wide; IX: 0.47 mm long, 0.21 mm wide; X: 0.48 mm long, 0.23 mm wide); XI is the longest, ~ 3.71 × longer than wide (0.78 mm long, 0.21 mm wide), subfusiform, with a blunt tip. Pilosity of antennomeres I–V comprised of short black setae, most decumbent though a few semi-erected, longer on segments I–III; antennomere VI with a mixture of short reddish brown and black setae; and antennomeres VII–XI with very short yellowish red setae, almost imperceptible.

Pronotum black, silky in appearance (Fig. 3A), small, sub-hexagonal, transverse, 1.59 × wider than long; length in the middle: 1.37 mm; maximum width (at the level of the lateral angles): 2.18 mm; lateral margins weakly converge backwards in the posterior two thirds and strongly converge forward in the anterior third, with the lateral angles well marked and rounded; fore margin almost straight; posterior margin or base broadly emarginated, with a thin flange. Dorsal surface of the pronotum clearly convex, gently sloping forward from the mid-region and steeply sloping back, with a slight and narrow depressed longitudinal-middle area with ambiguous boundaries (without a marked longitudinal midline or groove), such that two raised areas are observed on both sides of the central depression with two shallow and small rounded depressions observed anterior to the raised areas. Pronotal punctation relatively dense and unevenly distributed, consisting of relatively large, circular and deep punctures, subcontiguous, similar to those of the vertex but with a slightly larger diameter (Fig. 3A); the highest density is in the elevated areas on both sides of the midline, and the lowest densities are in the first quarter (just behind the fore margin), the depressed midband, and the central area of the base; integument surface with several fine, small and semi-wavy wrinkles between the punctures, located mainly on the sides, where they are arranged transversely and longitudinally in the middle depression. Pronotal setation inconspicuous, made up of short, curved dark reddish brown setae, mostly applied against the pronotal surface, distributed according to the pattern of punctures in which they are inserted; anterior margin, adjacent to the neck, with somewhat longer, semierect setae. Mesonotum mostly covered by the pronotum, showing, in dorsal view, only its posterior margin, which is weakly arcuate and with dense setation, consisting of setae longer than those of the pronotum, almost straight and lying backwards. Metanotum smooth, almost completely covered by the elytra. Prosternum narrow, very slightly extended posteriorly, broadly rounded at the central tip. Mesosternum relatively narrow and very transverse (width: 1.82 mm; length in the middle: 0.69 mm), with a small triangular prolongation backwards, ending in a rounded tip that extends to the level of the fore third of the mesocoxae; surface with long transverse wrinkles and dispersed punctures, similar to those of the vertex, and short setae. Metasternum subtrapezoidal, wide, covered by the mesocoxae, deep and closely notched in the middle of the posterior margin.

Elytra relatively short (length: 4.05 mm), strongly dehiscent and weakly convex, imbricated basally (the right over the left), divergent backwards and reaching the middle area of the fourth tergite, covering the first tergite, almost completely covering the second, and covering the lateral areas of the third (of which, only the central plate is clearly visible), and lateral basal portions on both sides of the fourth; elytral surface strongly rugose, corrugated, with marked wavy foveoles (Fig. 3C); punctation small, fine, shallow and scattered, confused with the roughness of the foveoles; integument with very dispersed and isolated setae, similar to those of the pronotum, although somewhat shorter.

Abdomen black, voluminous (Fig. 3C); maximum width, at level of the fourth tergite: 6.78 mm. Tergites semi-matte in appearance, with very weak and indistinct foveoles scattered on its surface; central sclerotised plates of the tergites elliptical, with a semi-glossy aspect and an integument surface that is slightly rough, with fine wrinkles, arranged transversely and concentrically. Dorsal setation decumbent, consisting of isolated and scattered short, reddish brown setae on the semi-matte sides of the tergites, and longer yellowish red (some almost golden) setae on the central plates, denser and forming inconspicuous tufts on the posterior margin of tergites II–IV. Ventrites silky in appearance, with dense punctation, made up of small, subcontiguous and slightly marked punctures that give them a finely vermiculated appearance; with short and decumbent dark brown, almost black, setae, homogeneously distributed; last ventrite clearly emarginated at the apex, with longer yellowish setae.

Legs relatively slender (Fig. 3C, D); surface with punctation fine and shallow, very dense in the tibiae and scarcer in the femurs, covered by relatively dense setation, consisting of short, dark brown (sometimes almost black) lying setae, denser on the tibiae. Length (in mm) of pro-, meso-, and metafemur as follows: 2.32, 2.6, and 3.1. Length (in mm) of pro-, meso-, and metatibia as follows: 2.25, 2.24 and 2.55. Length (in mm) of pro-, meso-, and metatarsus (and respective tarsomeres) as follow (claws excluded): 2.41 (I: 0.65; II: 0.4; III: 0.36; IV: 0.33; V: 0.67), 3.13 (I: 0.98; II: 0.53; III: 0.46; IV: 0.43; V: 0.73) and 3.56 (I: 1.36; II: 0.73; III: 0.61; IV: 0.86). Tarsi slender, clearly longer than the respective tibiae, with tarsomeres subcylindrical, slightly enlarged distally. Tarsomeres showing, on their ventral side, a small brush of very short, hirsute black setae, quite reduced in the last ones (V, V, IV). Pro- and mesotibiae with two similar distal spurs, short, narrow and straight; metatibial spurs dissimilar: outer spur spoon-shaped, inner spur similar to those of the fore- and mesotibiae but a little wider at the base and weakly curved at the apex. Coxae dense and finely punctate, with dense and short setation. Claws smooth, curved, with the lower lobe narrower than the upper one but equal in length.

Male genitalia with gonoforceps dark brown, hairless, moderately elongated, slender in dorsal, ventral, and lateral views (Fig. 4A–C). Gonostyli relatively long, ~ 4.4 × longer than wide in lateral view (1.33 mm long, 0.3 mm wide in lateral view), no excavated or depressed areas laterally in the distal regions; distal portion of each gonostylus separated dorsally by a fusiform longitudinal notch that extends to approximately the middle of the structure (Fig. 4A, B); distal lobes narrow and rounded at the apexes in lateral view (Fig. 4C). Gonocoxal plate relatively narrow and long, ~ 1.38 × longer than wide in dorsal view (1.36 mm long, 0.98 mm wide in ventral view), with the greatest width roughly in the middle of the plate, markedly emarginated at its distal margin (in ventral view) (Fig. 4A); surface almost flat. Aedeagus slender and narrow in lateral view (1.96 mm long, 0.2 mm wide in lateral view) flattened, narrowly rounded at the apex with two dorsal hooks that are similar in shape, although the distal hook is somewhat larger than the proximal one (Fig. 4D); endophallic hook small, located close to the apex and barely visible.

##### Variability.

Female similar to the male (Fig. 5) but with the last abdominal ventrite rounded and not emarginated in its posterior margin, and with relatively shorter antennae. Morphological measurements of the studied female specimens (paratypes): total length (frons to apex of tergite VIII): 10–14 mm (mean = 12 mm; *n* = 4); body length (frons to posterior border of elytra): 6.5–9.5 mm (mean = 8.3; n = 4); body maximum width (between the elytral external borders): 6–8.1 mm (mean = 7.4; *n* = 4); pronotum length: 1.6–1.8 mm (mean = 1.7; *n* = 4); pronotum maximum width: 2.23–2.74 mm (mean = 2.55 mm; *n* = 4); head maximum width: 2.74–3.43 mm (mean = 3.17 mm; *n* = 4); elytra length: 4–5.5 mm (mean = 5 mm; *n* = 4). Marked variability in the density of the pilose tufts on the dorsal side of the abdomen was observed: the studied females present lighter yellowish brown pilosity than that of the male, with more numerous and denser tufts located on the small, rounded depressed areas of the integument, homogeneously distributed, giving it an irregular appearance. Female gonostyli as in Fig. 4E.

##### Diagnosis and morphological comparisons.

Eurymeloe (B.) orobates is characterised morphologically, with respect to all the other species of the Bolognaia, by the following combination of diagnostic traits: (1) body size small or medium (total length: 10–14 mm); (2) body integument entirely black, semi-glossy in appearance; (3) setation of the head, pronotum and elytra, short and decumbent, reddish brown, moderately dark, sometimes very dark (almost black) ventrally and on the legs; (4) setation of the central plates of the abdominal tergites yellowish red (some almost golden), longer and forming inconspicuous tufts; (5) antennae slender and long, surpassing the base of the pronotum when extended backwards; (6) head broadly rounded, with a weak and relatively short longitudinal median groove; (7) pronotum small, very transverse (more than 1.5 × wider than long), sub-hexagonal; (8) pronotal surface showing a weak and narrow depressed longitudinal-middle area, but without a marked groove; (9) punctation of the head and pronotum dense, forming rounded and markedly deep punctures; (10) elytral surface strongly rugose, corrugated, with marked foveoles; and (11) male genitalia with long gonostyli with no excavated or depressed areas in the distal regions and a narrow and long gonocoxal plate.

The species most similar to *E.orobates* are *E.rugosus* and *E.apenninicus* (both belonging to the group B or *E.murinus* group). Both species present dark (black or dark brown) pilosity all over the body and, on the abdominal tergites, some inconspicuous (usually barely perceptible) yellowish brown or yellow setae, but not tufts. In addition to the colour pattern of the body setation, *E.rugosus* and *E.apenninicus* differ from *E.orobates* by the shape of their pronotum, which is longer, less transverse, and flatter (less convex), and has a strong median longitudinal groove (absent in *E.orobates*). The punctation of the head and pronotum are also markedly larger, deeper, and denser in *E.rugosus* and *E.apenninicus* (see Bologna 1988, 1991). In *E.orobates*, the antennae are slenderer and longer.

Within group B (*E.murinus* group), in which E. (B.) orobates is integrated, the new species can be readily distinguished from E. (B.) baudueri (southern France, Iberian Peninsula, and northern Morocco), E. (B.) flavicomus (Canary Islands), E. (B.) ganglbaueri (mainland Italy, Sardinia, Corsica, Sicily, Greece, Albania, Bulgaria, Bosnia and Herzegovina, Montenegro, Turkey, Syria, Spain, and southern France), E. (B.) gomari (northern Morocco), E. (B.) kandaharicus (Iran and Afghanistan), E. (B.) murinus (Iberian Peninsula, Sicily, Sardinia, Corse, Crete, Maghreb, and Libya), E. (B.) nanus (Iberian Peninsula, North Africa, and Middle East), E. (B.) omanicus (eastern Arabian peninsula), and E. (B.) pallidicolor (western Morocco). For instance, in contrast to E. (B.) orobates, all these species present, among other specific traits, a body integument that is dull grey or dark brown, occasionally reddish brown or, rarely, sandy brown (*E.pallidicolor*) or almost black (*E.ganglbaueri*). In addition, the body integument is generally opaque or matte in appearance or, at most, silky (but never glossy or semi-glossy as in *E.orobates*). The setation of these species is also quite distinct from that of *E.orobates*: it is yellowish, whitish, or golden all over the body and usually longer, and on the abdominal tergites, the tufts of setae, when present, are highly conspicuous (see Fig. 2). Moreover, the punctation of the head and pronotum in these species is clearly finer and shallower, and the elytral sculpture is distinctly smoother (not corrugated) and without marked foveoles, except in *E.ganglbaueri*; however, in this last species, the foveoles are clearly more attenuated than in *E.orobates* (see Kaszab 1958, 1983; Bologna 1988, 1991; Ruiz and García-París 2009, 2015).

Eurymeloe (B.) orobates differs from the species of group A (*E.mediterraneus* group, composed of, at least, *E.affinis* from the Maghreb and Libya; *E.apivorus* and *E.baamarani*, which are restricted to Morocco; *E.baudii* from the Italian Peninsula, Sicily, and Croatia; *E.glazunovi* from Eastern Europe and Central Asia; and *E.mediterraneus*, which is widely distributed throughout Europe, the Mediterranean basin, the Canary Islands, and the Middle East) by presenting reddish brown body setation and abdominal tergites with small tufts of reddish yellow setae, among other diagnostic characters (see above). By contrast, the body pilosity, including on the abdominal tergites, of species of the *E.mediterraneus* group is black (see Bologna 1988, 1991; Ruiz and García-París 2015). The Sardinian specimens of *E.mediterraneus* with brown setae can clearly be distinguished from *E.orobates* by the shape of the pronotum: in the first species, it is subrectangular and has subparallel sides; in the second, it is markedly transverse and subhexagonal and has sides that converge backwards.

The only species in group C is E. (B.) fernandezi (endemic to the Canary Islands). In comparison with E. (B.) orobates, this species presents, among other distinctive characters, an entirely black body setation; a clearly longer, not transverse pronotum with sinuous margins; an integument surface with wrinkles and parallel ridges that form eddies; and an elytral sculpture consisting of a fine zig-zag roughness (see Pardo Alcaide 1951; Ruiz and García-París 2015). Lastly, the two species tentatively assigned to *Bolognaia*, E. (B.) saharensis (widely distributed throughout North Africa, the Canary Islands, the Iberian Peninsula, Israel, and Saudi Arabia) and E. (B.) vignai (only known from Djibouti), are phenetically very different to E. (B.) orobates: their body setation is entirely reddish and longer, without reddish yellow setae forming tufts on the abdominal tergites; a subsquare, not transverse pronotum; relatively fine, shallow, and scattered punctation of the head and pronotum; very long legs; elytra with a soft sculpture, without foveoles or marked roughness; and highly distinctive male genitalia (Bologna 1988; Ruiz et al. 2010).

##### Distribution and notes on natural history.

**Figure 6.**
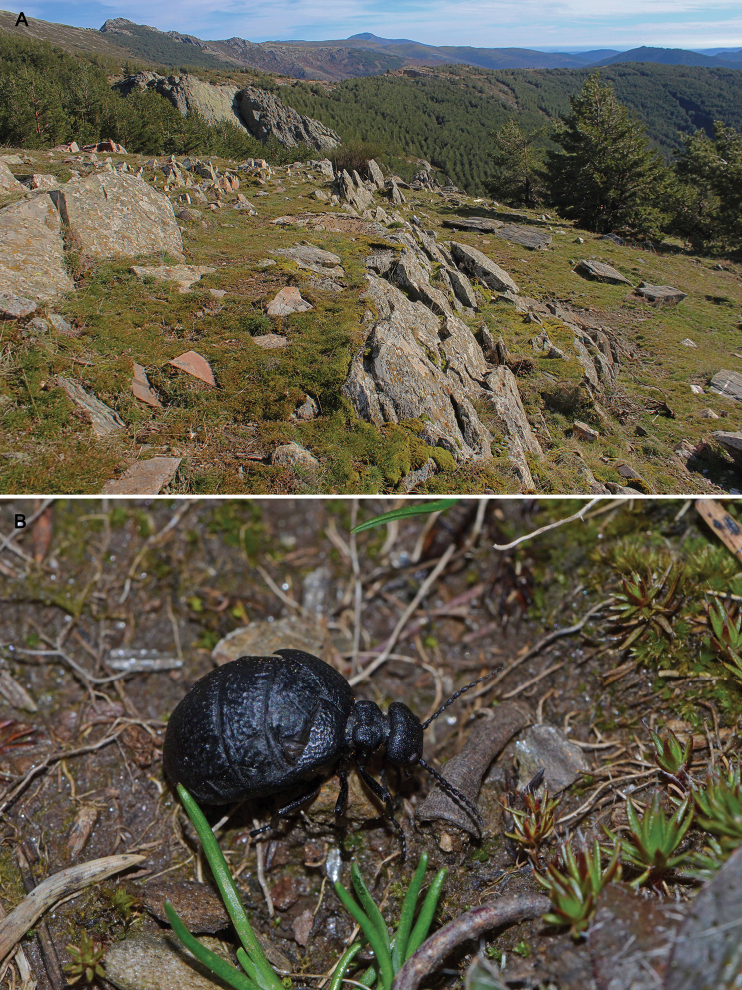
**A** Puerto de la Quesera, Guadalajara, Spain. Type locality of *Eurymeloeorobates* sp. nov. **B** adult female of *Eurymeloeorobates* sp. nov. in situ (paratype MNCN 325407). Photographs **A** (ASV) **B** (MGP).

*Eurymeloeorobates* is only known from a single locality, Puerto de la Quesera (in the province of Guadalajara, Spain) in the Iberian Peninsula (Fig. 6). This site, which is at an elevation of 1738 m above sea level (a.s.l.), is within the supra-Mediterranean bioclimatic level (see Rivas-Martínez 1987; Rivas-Martínez et al. 2002). Specifically, Puerto de la Quesera is in the Sierra de Ayllón, at the eastern edge of the Sistema Central mountain range. This region is characterised predominantly by micaceous schist, slate and quartzite soils (Rivas-Martínez et al. 1990; Vera 2004). Vegetation cover around Puerto de la Quesera consists of, at lower altitudes (below 1500 m a.s.l), deciduous oak forests of *Quercuspyrenaica* Willd. and, at higher altitudes (1500–1700 m a.s.l.), formations of *Fagussylvatica* L. Above the deciduous tree cover level, there are shrubs such as *Ericaarborea* L., *Juniperuscommunis* L., and *Arctostaphyllosuva-ursi* L., whereas grasslands dominate at altitudes over 1800 m a.s.l (Ibáñez et al. 1982). Hostile climatic conditions including low temperatures, late spring frosts, and strong winds characterise the high-altitude areas (Ibáñez et al. 1982). Furthermore, this region has been strongly altered by human activities (e.g., deforestation and overgrazing), particularly by the establishment of terraced pinewood plantations of *Pinussylvestris* L. (Fig. 6A) (Gil-García et al. 1995). In this region, adult specimens of *E.orobates* have been found actively wandering, under stones, and on tree barks, between November and May, usually in open areas or at the boundaries of the terraced plantations of *P.sylvestris* (authors, pers. obs.). Biological aspects of the new species remain unknown; however, we expect them to be similar to the ones described for other species of the *E.murinus* group (Bologna 1988, 1991).

##### Etymology.

The specific epithet *orobates* is derived from the Greek word “*oros*”, meaning mountain, and “*bates*”, meaning walker. This name alludes to the mountainous environment where the specimens of the new species were found, sometimes, wandering on mountain pastures and trails (Fig. 6B).

## ﻿Discussion

In light of previous morphological data and recent phylogenetic analyses (Sánchez-Vialas et al. 2021; this study), we have updated the internal taxonomy of the genus *Eurymeloe*. In order to reflect the morphologically distinguishable, main monophyletic units within *Eurymeloe*, and to maintain the validity of the widely used name *Coelomeloe*, we have deemed it necessary to consider that each of the three main molecular lineages represents an independent subgenus: *Eurymeloe*, *Coelomeloe*, and Bolognaia subgen. nov.

Morphological traits of larvae have been traditionally considered relevant in the systematics of the group, sometimes even more informative than adult characters for phylogenetic studies (Bologna and Pinto 2001). In fact, traits of the first instar larva (triungulin) have been studied for most of the genera and subgenera of Meloini (Bologna 1988, 1991; Selander 1989; Bologna et al. 1989, 1990; Bologna and Pinto 1992, 1998; Pinto and Bologna 1993; Bologna and Aloisi 1994; Di Giulio et al. 2002; Di Giulio et al. 2013, 2014). However, not having a resolved internal taxonomy for *Eurymeloe* confuses explanations of the evolutionary history of some of these traits. For instance, a particular morphological trait related to the shape of the abdominal spiracle I that is shared between the first instar larvae of *Coelomeloe* and *Eurymeloesensu stricto* [the *brevicollis* group of Meloe (Eurymeloe) sensuBologna 1988] was previously suggested to be the result of parallel biological adaptation (Di Giulio et al. 2013). However, considering our results and consistent with those shown by Sánchez-Vialas et al. (2021), this trait can be better explained as a synapomorphic character state for these sister subgenera.

Some conspicuous adult traits are also shared between the subgenera *Eurymeloe* and *Coelomeloe*, including antennae that are submoniliform, robust, short or medium in length, and which do not usually not reach the posterior margin of the pronotum; antennomeres V to VII that are wider than long or, at most, as wide as long; and very short or not [e.g., E. (E.) brevicollis, E. (E.) ibericus, and E. (C.) tuccia] body pubescence. These character states differ from those of the Bolognaia, which usually presents antennae that are moniliform, normally slender, long or medium in length, and which usually reach or exceed the posterior margin of the pronotum; antennomeres IV-IX that are subcylindrical, always longer than wide; and distinctive short or very short (black, yellowish, whitish, or golden) body pubescence. Therefore, the close relationship between *Eurymeloe* s. str. and *Coelomeloe* is supported by both our molecular analysis (BPP = 0.9) and morphology.

Our results confirm that E. (B.) rugosus, a species previously assigned to Bologna’s (1988) subgroup A (*E.rugosus* subgroup) on the basis of morphology, should instead be included in subgroup B (*M.murinus* subgroup). The morphology of *E.rugosus*, which presents a completely black coloration without noticeable brownish pilosity, led Bologna (1988) to separate it from subgroup B. Although both subgroups are now integrated within the Bolognaia, they are not monophyletic groups since, according to our molecular phylogeny, the morphological traits used to diagnose them are homoplastic. As a result, the assignation of some species to each of these groups has been controverted. For example, ambiguous morphological traits in *E.ganglbaueri* (see Ruiz & García-París 2009) has blurred its systematic allocation, as it was included in subgroup A based on morphology *sensu*Bologna (1988) but ascribed to that author’s subgroup B based on molecular data (Sánchez-Vialas et al. 2021). The newly discovered species, Eurymeloe (B.) orobates sp. nov., notably presents a pattern of pilosity that is intermediate between E. (B.) rugosus and E. (B.) murinus-E. (B.) ganglbaueri, but a body integument that is more similar to E. (B.) rugosus.

With the addition of the new species, eight species of the Bolognaia are known from the Iberian Peninsula: E. (B.) baudueri, E. (B.) ganglbaueri, E. (B.) mediterraneus, E. (B.) murinus, E. (B.) nanus, E. (B.) orobates, E. (B.) rugosus, and E. (B.) saharensis (García-París et al. 2010; Bologna, 2020a; this study). The existence of a new, morphologically distinctive species of Meloidae, which was found in an apparently well surveyed area of central Spain (Puerto de la Quesera, in the province boundaries between Madrid and Guadalajara), suggests that an undefined portion of the diversity of *Bolognaia* and other secretive species of *Eurymeloe* still awaits discovery.

## Supplementary Material

XML Treatment for Eurymeloe

XML Treatment for Bolognaia

XML Treatment for Eurymeloe

XML Treatment for Eurymeloe (Bolognaia) orobates
